# Cyp1b1 expression impacts the angiogenic and inflammatory properties of liver sinusoidal endothelial cells

**DOI:** 10.1371/journal.pone.0206756

**Published:** 2018-10-29

**Authors:** Juliana Falero-Perez, Yong-Seok Song, Yun Zhao, Leandro Teixeira, Christine M. Sorenson, Nader Sheibani

**Affiliations:** 1 Department of Ophthalmology and Visual Sciences, University of Wisconsin School of Medicine and Public Health, Madison WI, United States of America; 2 Deaprtment of Pathobiological Sciences, University of Wisconsin School of Veterinary Medicine, Madison, WI, United States of America; 3 Department of Pediatrics, University of Wisconsin School of Medicine and Public Health, Madison, WI, United States of America; 4 Department of Cell and Regenerative Biology, University of Wisconsin School of Medicine and Public Health, Madison, WI, United States of America; 5 Department of Biomedical Engineering, University of Wisconsin School of Medicine and Public Health, Madison, WI, United States of America; Medical College of Georgia at Augusta University, UNITED STATES

## Abstract

Cytochrome P450 1B1 (CYP1B1) is a member of the cytochrome p450 family of enzymes that catalyze mono-oxygenase reactions. Although constitutive Cyp1b1 expression is limited in hepatocytes, its expression and function in liver sinusoidal endothelial cells (LSEC) remains unknown. Here we determined the impact of Cyp1b1 expression on LSEC properties prepared from *Cyp1b1+/+* and *Cyp1b1-/-* mice. LSEC expressed PECAM-1, VE-cadherin, and B4 lectin similar to EC from other mouse tissues. *Cyp1b1* +/+ LSEC constitutively expressed significant levels of Cyp1b1, while *Cyp1b1-/-* LSEC lacked Cyp1b1 expression. LSEC also expressed VEGFR3, PROX-1, and LYVE-1, VEGFR1 and VEGFR2, as well as other cell adhesion molecules including ICAM-1, ICAM-2, VCAM-1, and thrombospondin-1 (TSP1) receptors, CD36 and CD47. However, the expression of PV-1 and stabilin (fenestration markers), and endoglin were limited in these cells. The *Cyp1b1-/-* LSEC showed limited fenestration, and decreased levels of VEGF and BMP6. *Cyp1b1-/-* LSEC also showed a decrease in the levels of VE-cadherin and ZO-1 impacting adherens and gap junction formation. *Cyp1b1-/-* LSEC were significantly more apoptotic, proliferated at a faster rate, and were less adherent and more migratory. These changes were attributed, in part, to decreased amounts of TSP1 and increased AKT and ERK activation. The expressions of integrins were also altered by the lack of *Cyp1b1*, but the ability of these cells to undergo capillary morphogenesis was minimally affected. Furthermore, *Cyp1b1-/-* LSEC expressed lower levels of inflammatory mediators MCP-1 and TNF-α. Thus, *Cyp1b1* expression has a significant impact on LSEC angiogenic and inflammatory functions.

## Introduction

The hepatic sinusoids are covered with blood vessels that perfuse the hepatocytes. They serve as a location for the oxygen-rich blood from the hepatic artery and the nutrient-rich blood from the portal vein, and transport blood from the porta hepatis to the inferior vena cava through the liver [1]. Liver sinusoidal endothelial cells (LSEC) are highly specialized and line the hepatic sinusoidal wall [2–4]. They are one of the first hepatic cell population that come to contact with blood, separating blood in the sinusoid from the extracellular space of Disse and surrounding hepatocytes [5–7]. Although LSEC number represent a small percentage of all liver cells [8–10], they have specific and important physiological functions that are not yet fully appreciated.

LSEC participate in the endocytosis and metabolism of a wide range of macromolecules [8], and are in intimate contact with leukocytes passing through the liver [11]. LSEC together with macrophages and hepatocytes take up liposomes through direct recognition of phospholipid head groups by the scavenger receptors expressed on their cell surface [12]. LSEC in combination with Kupffer cells constitute the most powerful scavenger system in the body [13, 14]. LSEC also play a key role in the regulation of iron homeostasis by expression of bone morphogenic protein 6 (BMP6) and the production of iron regulatory hormone, hepcidin, by hepatocytes [15].

LSEC are an important component of the complex network of cellular interactions, which cooperate to sustain liver function [8]. They facilitate and regulate the bi-directional transfer of substrates between the blood and liver parenchyma, forming a blood-hepatocyte barrier [16, 17]. In order to maximize the transfer of substrates between blood and hepatocytes, LSEC exhibit a unique morphology with cytoplasmic extensions that are very thin and perforated with pores called fenestrations [18]. Fenestrations are specialized plasma membrane micro-domains appearing as circular discontinuities of 50–200 nm in diameter [19, 20]. There are approximately 3–20 fenestrations per μm^2^ of LSEC surface defining them as an ultrafiltration system [18]. Fenestrations change dynamically in frequency and diameter in response to numerous stimuli in vivo and in vitro. Small changes in fenestrations have profound effects on the size and number of macromolecules passing through the liver sinusoidal endothelium [8, 20]. Fenestrations respond to various stimuli such as inflammation, dietary fat load, circulating vasoactive cytokines and hormones [9]. Decreased fenestration (defenestration) occurs in aging and various diseases [21, 22] resulting in increased hepatic lipoprotein deposition [23].

Vascular endothelial growth factor (VEGF) is an important regulator of angiogenesis and vascular permeability [24]. It is continuously expressed in epithelial cells of adult organs with fenestrated endothelium, such as choroid plexus and kidney glomeruli [25], and is sufficient to induce fenestration [26, 27]. Paracrine production of VEGF is involved in the induction and/or maintenance of fenestrations in adjacent EC expressing VEGF receptors [25, 26, 28]. In addition, the application of VEGF in vivo can directly and rapidly induce fenestrae in the continuous endothelium of skeletal muscle and skin [29], and in the neovasculature of VEGF-secreting tumors [30]. Thus, VEGF is an essential factor for regulation of fenestrations.

Cytochrome P450s expression are vital to the detoxification activity of liver hepatocytes. However, the contribution of these Cyps in the liver vasculature to these activities are just emerging. Cyp1b1 is a member of Cyp superfamily with important metabolic activity. Although constitutive Cyp1b1 expression in hepatocytes is limited [31, 32], its expression in liver vasculature and its contribution to various liver functions remains largely unknown. We recently showed Cyp1b1 expression is essential during angiogenesis and in proper vascular cell function [33–36]. The absence of Cyp1b1 was associated with increased oxidative stress, sustained activation of NF-κB, and upregulation of the angioinhibitory protein thrombospondin-2 in retinal vascular cells. In addition, all changes were reversed by the addition of N-acetylcysteine or NF-κB inhibitors [33, 35], establishing an important role for Cyp1b1 in cellular redox homeostasis.

Cyp1b1 deficiency was recently shown to suppress hepcidin expression in the liver [37], a gene whose expression is regulated by the BMP6 produced by LSEC [38, 39]. Here we tested the hypothesis that *Cyp1b1* is constitutively expressed in LSEC, and its expression has a significant impact on their properties. To address this hypothesis we isolated LSEC from *Cyp1b1+/+* and *Cyp1b1-/-* mice. We showed increased apoptosis rate in *Cyp1b1-/-* LSEC compared to *Cyp1b1+/+* cells that is compensated in part by increased proliferation. Using electron microscopy, we revealed decreased fenestration in *Cyp1b1-/-* LSEC. This was likely associated with decreased production of VEGF in *Cyp1b1-/-* LSEC. Lack of *Cyp1b1* had a significant impact on migration, proliferation and adhesion of LSEC. These changes were attributed, in part, to decreased levels of TSP1 and fibronectin, and altered expression of integrins in these cells. In addition, *Cyp1b1*-*/-* LSEC expressed a significantly lower level of ICAM-1, BMP6, and proinflammatory cytokines MCP-1 and TNF-α. Thus, expression of *Cyp1b1* in LSEC affects their angiogenic and inflammatory properties.

## Materials and methods

### Ethics statement

All animal experiments were performed in accordance to the Association for Research in Vision and Ophthalmology Statement for the Use of Animals in Ophthalmic and Vision Research and were approved by the Institutional Animal Care and Use Committee of the University of Wisconsin School of Medicine and Public Health (the assurance number A3368-01). Animals were sacrificed according to an approved protocol by CO2 asphyxiation.

### Animals

For liver sinusoidal endothelial cell (LSEC) isolation, Immorto *Cyp1b1+/+* and *Cyp1b1-/-* mice expressing a temperature-sensitive SV40 large T antigen (Charles River Laboratories, Wilmington, MA) in a C57BL/6j background were generated as previously described [33].

### Isolation and culture of LSEC

LSEC were isolated from one litter (2–3 pups) of 4-week-old *Cyp1b1+/+* and *Cyp1b1-/-* Immorto mice by collection of livers under a dissecting microscope. Livers were rinsed with serum-free Dulbecco’s Modified Eagle’s medium (DMEM), minced into small pieces with a sterilized razor blade in a 60-mm tissue culture dish, and digested with 2 ml of collagenase type I (1 mg/mL in serum-free DMEM; Worthington, Lakewood, NJ) at 37°C. After digestion, cells were rinsed with 5 ml of DMEM containing 10% fetal bovine serum (FBS), centrifuged for 5 min at 400 xg, and resuspended in 5 ml of DMEM with 10% FBS. After filtration through a double layer of sterile nylon mesh (40 μm; Sefar America, Fischer Scientific, Hanover Park, IL), cells were centrifuged at 400 xg for 10 min and rinsed twice with DMEM containing 10% FBS. Cells were re-suspended in 1 ml of DMEM with 10% FBS and incubated with magnetic-beads coated with anti-PECAM-1 antibody for 1 h at 4°C on a rocker as previously described [40]. After incubation, cells bound to the magnetic beads were collected with a magnetic tube holder and washed six times with 1ml of DMEM containing 10% FBS, and bound cells were plated in a single well of a 24-well plate coated with fibronectin (2 μg/ml in serum-free DMEM; BD Biosciences, Bedford, MA) in 0.5 ml of EC growth medium and incubated in a tissue culture incubator at 33°C and 5%CO_2_. EC were grown in DMEM containing 10% FBS, 20 mM HEPES, 2 mM sodium pyruvate, 1% nonessential amino acids, 100 μg of streptomycin, 2 mM L-glutamine, 100 U/ml penicillin, 55 U/ml heparin (Sigma), 100 μg/ml of EC growth supplement (Sigma), and 44 U/ml recombinant murine interferon-γ (R&D Systems). Cells were incubated at 33°C with 5% CO_2_ and progressively passaged to larger plates and maintained on 1% gelatin-coated 60-mm tissue culture dishes. For all the experiments performed here at least two different isolation of LSEC were used.

### FAC scan analysis

Cell surface expression of various proteins were determined by flow cytometry as previously described [40]. The following primary antibodies were used: anti-PECAM-1 (553370; BD Biosciences), anti-VE-cadherin (ALX-210-232-C100; Enzo Life Sciences), B4-lectin (FL1201,Vector Labs), anti-ICAM-1 (553250; BD Biosciences) and anti-ICAM-2 (553326; BD Biosciences), anti-VCAM-1 (CBL1300; Millipore), anti-CD36 (552544; BD Bioscience), anti-CD47 (1407882; eBioscience), anti-LYVE-1 (MAB2125; R&D systems), anti-PROX-1 (11067-2-AP; Proteintech), anti-VEGFR1 (MAB471; R&D systems), anti-VEGFR2 (MAB443; R&D systems), anti-VEGFR3 (552857; BD Bioscience), anti-PV-1 (550563; BD Bioscience), anti-stabilin (SC27751; Santa Cruz), anti-endoglin (550546; BD Bioscience), anti-α1 (555001, BD Biosciences); anti-α2 (558295; BD Biosciences); anti-α3 (AB1920; Millipore); anti-α4 (AB1924; Millipore); anti-α5 (AB1921; Millipore); anti-α6 (MAB1378; Millipore), anti-β1 (MAB 2000; Millipore), anti-β5 (SC-5401; Santa Cruz), anti-β8 (SC-25714; Santa Cruz), anti-α5β1 (MAB1999), anti-α6 (MAB1378), anti-αv (AB1930), anti-αvβ3 (MAB1976), and anti-α3 (MAB1957; all from Millipore) integrins at dilutions as recommended by the suppliers. Following incubation with primary antibodies, cells were washed with Tris-buffered saline (10 mM Tris-HCl, 150 mM NaCl, pH 7.6; TBS, containing 1% BSA) and incubated with appropriate FITC-conjugated secondary antibodies (Jackson Immunoresearch Laboratories, Inc., West Grove, PA). Following incubation, cells were washed with TBS with 1% BSA twice, resuspended in TBS with 1% BSA and analyzed by FACScan caliber cytometer (Becton-Dickson, Franklin Lakes, NJ).

### Electron microscopy

The electron microscopy samples were prepared as previously described [41]. Liver EC of very early passage (3–4) were cultured on fibronectin-coated cover slips, collected, fixed, embedded and were sectioned. Ultrathin sections (90 nm) were cut, mounted, and assessed using a Philips 410 transmission electron microscope (Philips Medical System, Andover, MA). Images were captured in digital format.

### VEGF analysis

VEGF protein levels produced by *Cyp1b1+/+* and *Cyp1b1-/-* LSEC were determined using a mouse VEGF Immunoassay kit (R&D Systems). Cells were plated at 8×10^5^ on 60 mm tissue culture dishes in EC growth medium. After 24 h, the cells were rinsed once with serum-free DMEM and incubated with 2 ml of serum-free DMEM for another 48 h. The conditioned medium was collected, centrifuged for 5 min at 400 xg to remove cell debris, and the secreted VEGF was analyzed per manufacturer’s instructions. The amount of VEGF was determined using a standard curve generated with known amounts of VEGF in the same experiment.

### Immunofluorescence analysis

Cells (5x10^4^) were plated on fibronectin-coated glass chamber slides until confluent (1–2 days), washed in 1x MES (M5287; Sigma), fixed with 4% paraformaldehyde and permeabilized with 0.1% triton X100 for 10 min on ice, and blocked with 1% ovalbumin in TBS at 37°C for 20 min. Slides were washed with PBS and incubated with: anti-VE-cadherin (550548; BD Biosciences), anti-N-cadherin (610920; BD Biosciences), anti-β-catenin (610154; BD Biosciences), anti-P-120 catenin (610133; BD Biosciences), anti-ZO-1 (617300; Invitrogen), anti-vinculin (V4505; Sigma), and Alexa Fluor 488 Phalloidin (A12379; Thermo Fisher) in TBS containing 1% ovalbumin at 37°C for 30 min. After washing with PBS, cells were incubated with appropriate Cy3-conjugated secondary antibody (Jackson ImmunoResearch; 1:500 dilution in TBS containing 1% ovalbumin) at 37°C for 30 min. Cells were washed with PBS twice, analyzed using a fluorescent microscope (Carl Zeiss Optical Inc., Germany), and images were captured in digital format.

### Western blot analysis

Cell lysates were prepared and analyzed by 4–20% SDS-PAGE as previously described [33]. Proteins were transferred to nitrocellulose membranes and blotted with specific antibodies: anti-CYP1B1 (a rabbit polyclonal antibody developed in Dr. Colin Jefcoate’s laboratory, University of Wisconsin), anti-VE-cadherin (ALX-210–232-C100; Enzo Life Sciences), anti-N-Cadherin (610920; BD Biosciences), anti-β-catenin (610154; BD Biosciences), anti-P120- catenin (610133; BD Biosciences), anti-ZO-1 (617300; Invitrogen), anti-thrombospondin-1 (MS-421P; Neo Markers, Fremont, CA), anti-thrombospondin-2 (611150; BD biosciences), anti-osteonectin (AF942; R&D Systems), anti-osteopontin (AF808; R&D Systems) and anti-tenascin-C (AB19013; Millipore), anti-Akt (9272; Cell Signaling), anti-pAkt (9271; Cell Signaling), anti-JNK (AF1387; Cell Signaling), anti-pJNK (9271; Cell Signaling), anti-ERK (9102; Cell Signaling), anti-p-ERK (9106; Cell Signaling), anti-Src (2123p; Cell Signaling), anti-p-Src416 (2101; Cell Signaling), anti-p38 (9212; Cell Signaling), anti-p-p38 (9211; Cell Signaling), anti-p-eNOS (7570, Cell Signaling) and anti-eNOS (32027; Cell Signaling). All blots were stripped and incubated with anti-β-actin (Sigma) antibody for loading control. For analysis of secreted proteins, cells were incubated with serum free growth medium for 48 h. Conditioned medium was collected, clarified by centrifugation, and the level of various ECM proteins was determined by Western blot analysis as described above.

### Cell proliferation assay

Cell proliferation assay was performed by counting the number of cells every other day for 2 weeks. Cells (1X10^4^) were plated on multiple sets of gelatin-coated 60 mm tissue culture dishes, and the cells were counted the next day for day one. Cells were then fed every other day and counted on the days that they were not fed.

### Cell viability assays

Cells of each genotype were plated at 1.5x10^4^ per well of a 96 well plate overnight. The next day, cells were incubated with 1.4 mM of hydrogen peroxide (H_2_O_2_) (Fisher Scientific, Fair Lawn, NJ) for 24 h at 33°C. The percentage of live cells was determined using the CellTiter 96 AQ_ueous_ Non-Radioactive Cell Proliferation Assay kit (Promega, Madison, WI). These experiments were repeated with two different isolation of LSEC with similar results.

### Apoptosis assays

*Cyp1b1+/+* and *Cyp1b1-/-* LSEC were plated at 1.25x10^4^/well in a 96 well plate. The next day, cells were incubated with 1.4 mM H_2_O_2_ for 8 h and compared with basal level of apoptosis. Caspase-3 and -7 activities of apoptotic cells were evaluated by a Caspase-Glo 3/7 assay (Promega, Madison, WI) using a luminescent microplate reader (Victa2 1420 Multilabel Counter, PerkinElmer, Waltham, MA).

### Scratch wound assays

Cells (8×10^5^) were plated in 60-mm tissue culture dishes and allowed to reach confluence (2–3 days). Cell monolayers were wounded using a 1 ml micropipette tip, rinsed twice with DMEM containing 10% FBS, and fed with EC growth medium containing 1 μM 5-fluorouracil (Sigma) to exclude potential contribution of cell proliferation to wound closure. The wound closure was monitored and photographed at 0, 24, 48 and 72 h with a phase microscope in digital format. For quantitative assessment, the distances migrated as percentage of total distance were determined.

### Transwell migration assays

Prior to the assay, cells were incubated in serum-free EC growth medium for 24 h. Transwell filters (Corning, Acton, MA) were coated with 2 μg/ml fibronectin in serum free DMEM and incubated overnight at 4°C. The bottom of the transwell was rinsed with PBS and blocked with 2% BSA in PBS for 1h at room temperature. The transwell was further rinsed with PBS and 500 μl serum-free DMEM was added to the bottom of each well. Cells, suspended at 1×10^5^ in 100 μl of serum-free medium, were added to the top of the transwell membrane. Cells were incubated for 3 h at 33°C, fixed with 2% paraformaldehyde (PFA) for 10 min at room temperature, and stained with hematoxylin and eosin. The stained membranes were mounted on a glass slide, and the number of migrated cells through the membrane, which attached to the bottom, were determined by counting 10 high-power fields (×200).

### Cell adhesion assays

Cell adhesion to various ECM proteins were performed as previously described [42]. Briefly, cells of each genotype were plated into wells of a 96-well plate previously coated with various concentrations of ECM proteins including fibronectin, vitronectin, collagen I and Collagen IV (BD biosciences). Following 2 h of incubation, cells that were not able to adhere were washed away until no cells left in control wells coated with BSA. Attached cells were then lysed and quantified for intracellular acid phosphatase levels using a 96-well plate reader at 405 nm.

### Capillary morphogenesis

Tissue culture plates (35 mm) were coated with 0.5 ml of Matrigel (10 mg/ml, BD Bioscience) and allowed to harden by incubating at 37°C for at least 30 min. Cells were removed by trypsin EDTA, washed with DMEM containing 10% FBS, and resuspended at 2x10^5^ cells/ml in growth medium without FBS. Cells (2x10^5^) in 2 ml were applied to the Matrigel-coated plates, incubated at 33°C, photographed after 18 h using a Nikon microscope in a digital format. For quantitative assessment of the data, the mean numbers of branch points were determined by counting the number of branch points in five high-power fields (x100). Longer incubation times did not further improve the degree of capillary morphogenesis.

### Quantitative real time PCR (qPCR) analysis

Total RNA from *Cyp1b1+/+* and *Cyp1b1-/-* LSEC was extracted using mirVana PARIS kit (Invitrogen). The cDNA synthesis was performed from 1 μg of total RNA using Sprint RT Complete-Double PrePrimed kit (Clontech, Mountain View, CA). One μl of each cDNA (dilution 1∶10) was used as template in qPCR assays, performed in triplicate of three biological replicates on Mastercycler Realplex (Eppendorf) using the SYBR-Green qPCR Premix (Clontech). Amplification parameters were as follows: 95°C for 2 min; 40 cycles of amplification (95°C for 15 sec, 60°C for 40 sec); dissociation curve step (95°C for 15 sec, 60°C for 15 sec, 95°C for 15 sec). Primer sequences used Cyp1b1 5′-CCAGGCGTCGCACTTGTACT-3’ (forward) and Cyp1b1 5’-TGGAAAACGTC GCCATAGC-3’(reverse). For VEGF164 5’-GCAGCTTGAGTTAAACGAACG-3’(forward) and VEGF164 5’-GGTTCCCGAAAC CCTGAG-3’(reverse); Bmp6 5’-GTGACACCG CAGCACAAC-3’(forward); Bmp6 5’-TCGTAAGGGCCGTCTCTG-3’ (reverse); Mcp1 5’-GTCTGTGCTGACCCCAAGAAG-3’(forward) and Mcp1 5’-TGGTTCCGATCC AGGTTTTTA-3’(reverse); IL-1β 5’-GTTCCCATTAGACAAC TGCACTACA-3’ (forward) IL-1β: 5’-CCGACAGCACGAGGCTTTT-3’ (reverse); IL- 6 5’- CAACCACGGCCTTC CCTACT-3’(forward) and IL-6 5’-TTGGGAGTGGTATCCTCTGTGA-3’ (reverse); and for Tnf-α 5’- ACCGTCAGCCGATT TGCTAT-3’(forward) and Tnf-α 5’-TTGACGGCAGA GAGGAGGTT-3’(reverse). Standard curves were generated from known quantities for each of the target gene of linearized plasmid DNA. Ten times dilution series were used for each known target, which were amplified using SYBR-Green qPCR. The linear regression line for ng of DNA was determined from relative fluorescent units (RFU) at a threshold fluorescence value (Ct) to quantify gene targets from cell extracts by comparing the RFU at the Ct to the standard curve, normalized by the simultaneous amplification of RpL13a, a housekeeping gene. Primer sequences for RpL13a were 5′-TCTCAAGG TTGTTCG GCTGAA-3′ (forward) and Rpl13a 5′-CCAGACGCCCCAGGTA-3′ (reverse).

### Statistical analysis

Statistical differences between groups were evaluated with the One-way ANOVA followed by Tukey’s multiple comparison test using GraphPad Prism version 5.04 for Windows (GraphPad Software, La Jolla, CA). Statistical Differences were confirmed with Bonferroni’s comparison of selected pairs of columns and student’s unpaired t-test (two-tailed). Mean ± standard deviation is shown. P< 0.05 is considered significant.

## Results

### Isolation of mouse LSEC

**Fig 1:**
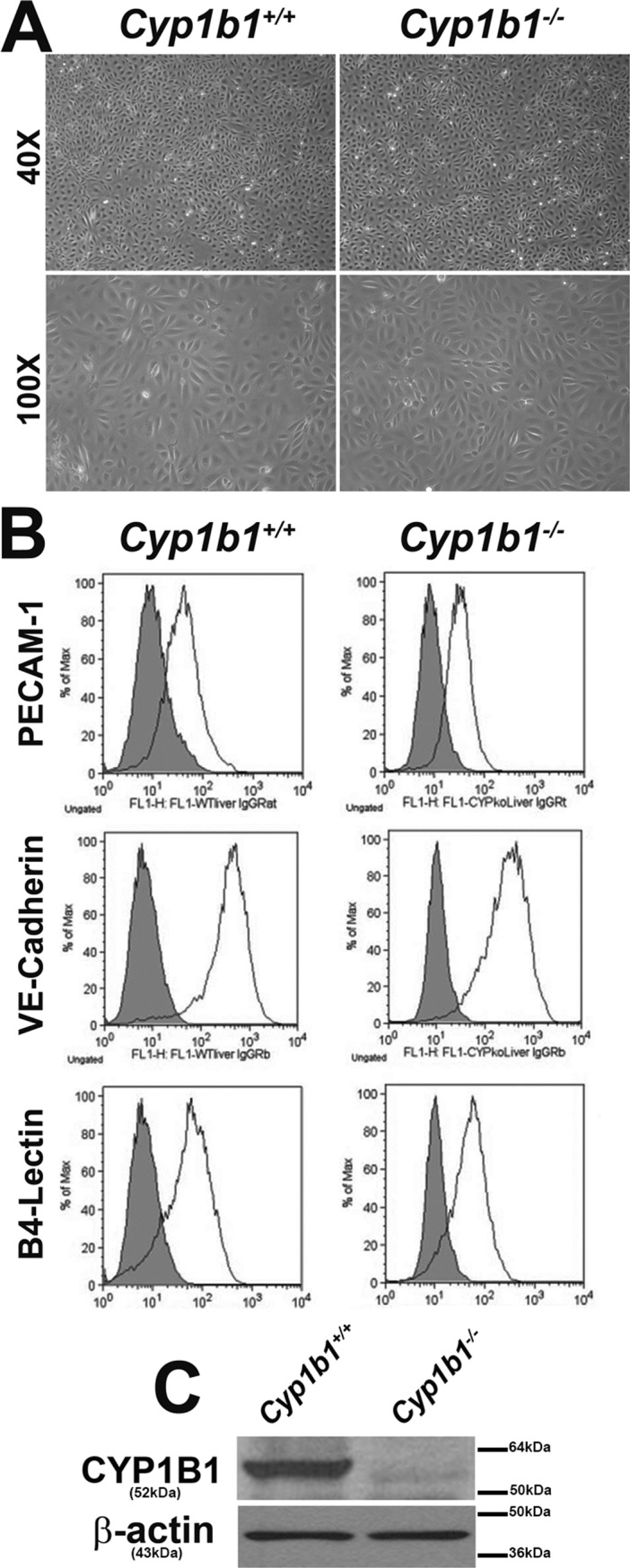
Isolation of mouse LSEC. *Cyp1b1*+/+ and *Cyp1b1-/-* LSEC were isolated and cultured on gelatin-coated 60-mm dishes as detailed in Methods. (A) Cells were photographed in digital format at ×40 and ×100 magnifications. Please note no differences in the morphology of these cells. (B) Expressions of vascular EC markers in LSEC. LSEC were examined for expression of PECAM-1, VE-Cadherin and B4-Lectin by FACS analysis. Shaded area show control IgG staining. (C) Expression of Cyp1b1 in LSEC by western blotting. β-actin was used as a loading control. These experiments were repeated with 3 isolations of LSEC with similar results.

We previously showed Cyp1b1 expression is essential during angiogenesis and proper retinal vascular cell function [33–36]. However, the expression of Cyp1b1 in LSEC, a fenestrated type of EC, has not been previously determined. Here we wished to determine whether LSEC constitutively express Cyp1b1, and how Cyp1b1 expression affects their function. Isolation of LSEC from various species have been previously reported [43–48]. These cells have provided a valuable tool for the study of liver pathophysiology and the role these cells play in liver function. We prepared LSEC from *Cyp1b1*+/+ and *Cyp1b1-/-* Immortomice. Fig 1A shows the morphology of *Cyp1b1*+/+ and *Cyp1b1-/-* LSEC. They are both very similar to LSEC that are widely acknowledged [43, 49]. Please note that lack of *Cyp1b1* expression minimally affected cell morphology.

Classic mouse vascular EC commonly express PECAM-1, VE-Cadherin and B4-Lectin [40]. To further confirm the identity of these cells as EC, we determined the expression of these markers. FACS analysis confirmed positive expression of these markers in *Cyp1b1*+/+ and *Cyp1b1-/-* LSEC (Fig 1B). Please note that the PECAM-1 expression profile here agrees with the general understanding that LSEC express lower levels of PECAM-1 than continuous vascular EC [6].

**Fig 2:**
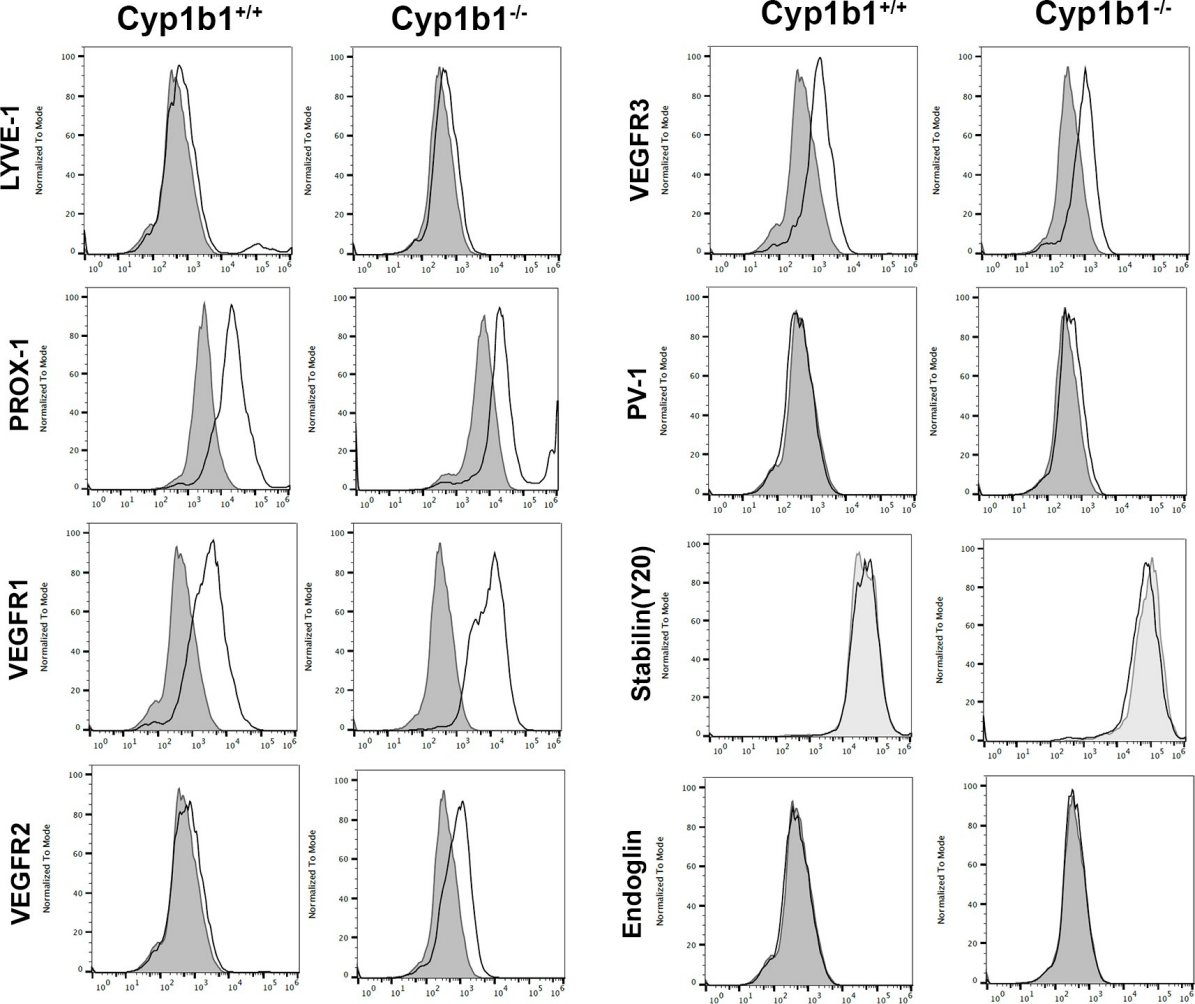
LSEC express other EC markers. The expression LYVE-1, PROX-1, VEGFR1, VEGFR2, VEGFR3, PV-1, Stabilin, and endoglin were determined by FACS analysis of *Cyp1b1*+/+ and *Cyp1b1-/-* LSEC. Please note the increased levels of VEGFR2 in *Cyp1b1-/-* LSEC. These experiments were repeated with two different isolation of LSEC with similar results.

**Fig 3:**
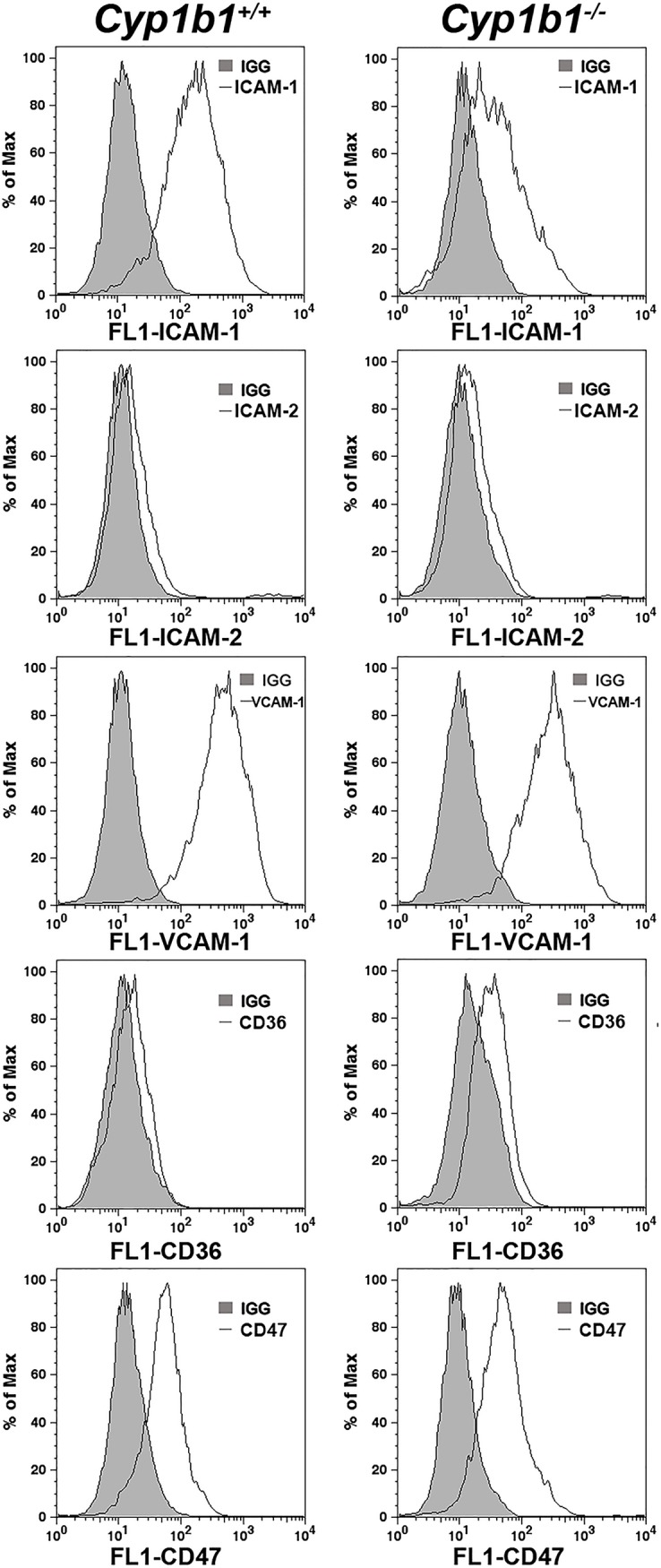
Expression of EC adhesion molecules in LSEC. The expression of ICAM-1, VCAM-1, CD36, and CD47 were determined by FACS analysis of *Cyp1b1*+/+ and *Cyp1b1-/-* LSEC. Please note a dramatic decrease in the level of ICAM-1 in *Cyp1b1-/-* LSEC as compared with wild type cells. Levels of ICAM-2, VCAM-1 and CD47 remained similar between the two cell types. Expression of CD36 was modestly increased *Cyp1b1-/-* LSEC. These experiments were repeated with two isolations of LSEC with similar results.

The expression of CYP1B1 was assessed by Western blotting in the cell lysates prepared from *Cyp1b1*+/+ and *Cyp1b1-/-* LSEC. Mouse LSEC constitutively expressed CYP1B1 (Fig 1C). As expected, the expression of CYP1B1 was not detected in the *Cyp1b1-/-* cells. Thus, mouse LSEC constitutively express CYP1B1. Interestingly, neither *Cyp1b1*+/+ or *Cyp1b1-/-* LSEC showed detectable expression of CD105 (Fig 2), a critical molecule in classic and continuous vascular development [50]. Fig 2 also shows expression of additional LSEC markers including LYVE-1, PROX-1, VEGFR1, VEGFR2, VEGFR3, and fenestration makers PV-1 and stabilin. Both Cyp1b1+/+ and Cyp1b1-/- LSEC expressed detectable levels of LYVE-1 and PROX-1, markers of lymphatic EC. These cells also expressed similar level of VEGFR1 and VEGFR3. The level of VEGFR2 was higher in *Cyp1b1-/-* cells compared to *Cyp1b1*+/+ cells. We detected minimal levels of fenestration marker proteins PV-1 and stabilin. Fig 3 shows the expression of other cell adhesion proteins including ICAM-1, ICAM-2, VCAM-1, CD36, and CD47. Although both cells expressed similar levels of ICAM-2, the level of ICAM-1 was decreased in *Cyp1b1 -/-* LSEC. Both cell types expressed similar levels of VCAM-1 and CD47. The expression of scavenger receptor CD36 was, however, higher in *Cyp1b1-/-* LSEC.

### Loss of fenestrations and decreased VEGF production in Cyp1b1-/- LSEC

**Fig 4:**
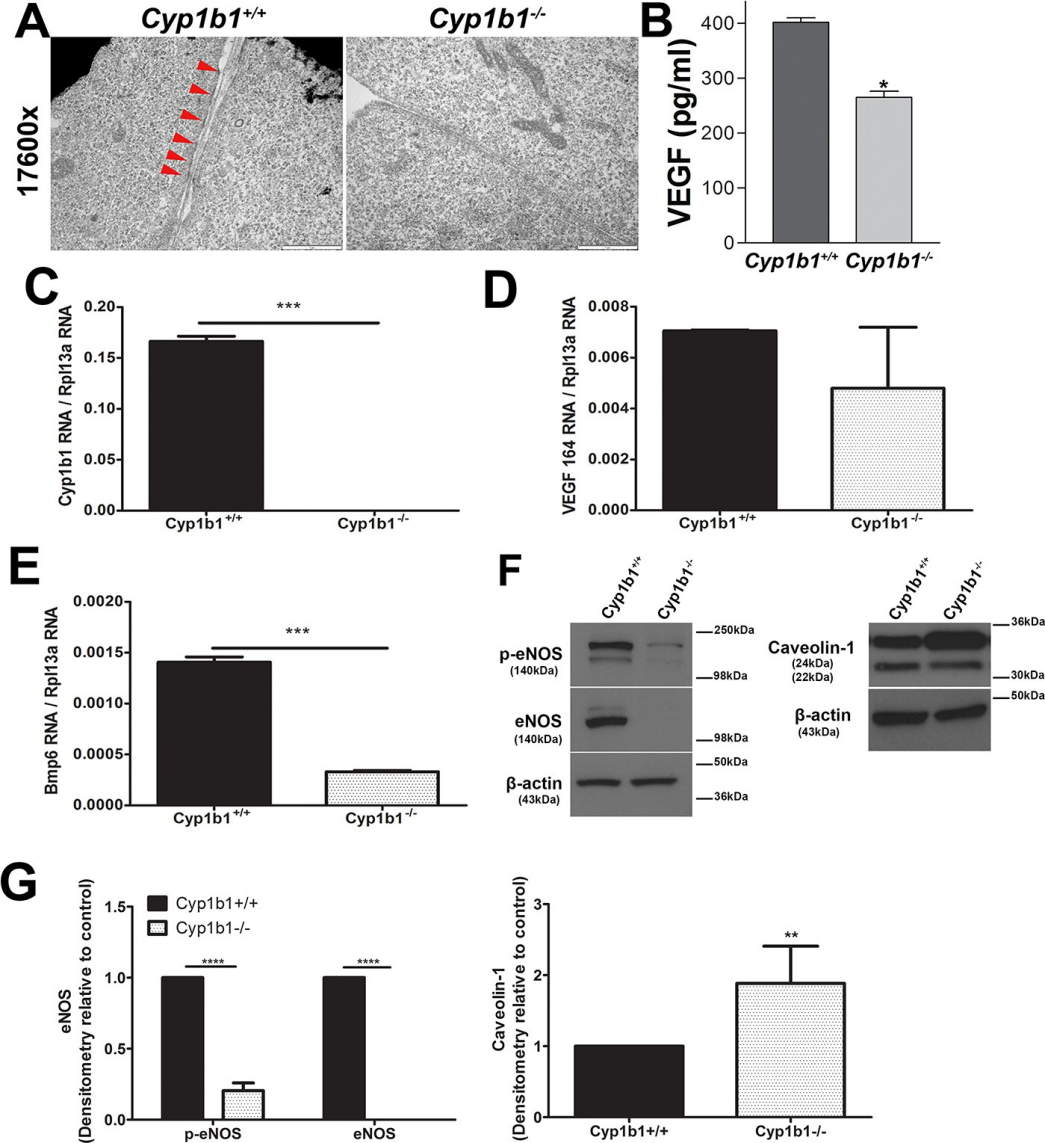
Loss of fenestrations and decreased VEGF production in *Cyp1b1-/-* LSEC. (A) *Cyp1b1*+/+ and *Cyp1b1-/-* *LSEC* of early passage were examined under the electron microscopy (×17600) for fenestrations. Clusters of fenestrations were found aligned in the peripheries of *Cyp1b1*+/+ LSEC with the expected size of 50–200 nm in diameter [19, 20] (left, arrowheads). In contrast, no fenestration structures were found in *Cyp1b1* -/-LSEC (right). (B) A significant decrease in the secretion of VEGF was observed in *Cyp1b1-/-* LSEC compared with *Cyp1b1*+/+ cells (*p<0.05, n = 3). We also confirmed the expression of Cyp1b1 (C), VEGF164 (D), and BMP6 (E) in these cells by qPCR analysis. Please note the absence of Cyp1b1 expression in null cells. No significant difference was observed in the mRNA levels of VEGF. A significant decrease in the amounts of Bmp6 mRNA was observed in *Cyp1b1-/-* LSEC (***P< 0.001; n = 3). Expression of eNOS, phospho-eNOS (p-eNOS) and caveolin-1 was determined by Western blot analysis of lysates prepared from *Cyp1b1*+/+ and *Cyp1b1-/-* LSEC (F). The quantitative assessment of the data is shown in (G). The β-actin was used as loading control. Please note a significant decrease in the levels of eNOS and p-eNOS in *Cyp1b1-/-* LSEC (****P< 0.0001; n = 3). A significant increase in the level of caveolin-1 α-isoform and a decrease in the caveolin-1 β-isoform were noted (**P< 0.01; n = 3). These experiments were repeated with two isolation of LSEC with similar results.

In order to evaluate the filtration function of LSEC upon *Cyp1b1* deficiency, fenestrations of *Cyp1b1+/+* and *Cyp1b1-/-* LSEC were evaluated using electron microscopy. The fenestrations of *Cyp1b1+/+* LSEC were frequently found in the cellular plasma membrane (Fig 4A, left, arrowheads). In contrast, no such fenestration could be detected in *Cyp1b1-/-* LSEC (Fig 4A, right). Since VEGF is a critical regulator of fenestration formations, we further compared the levels of VEGF that was secreted by the two cell types. A 33% decrease in VEGF production was observed in *Cyp1b1-/-* LSEC compared with *Cyp1b1+/+* cells (Fig 4B). Fig 4C shows the mRNA expression of *Cyp1b1* in these cells and as expected *Cyp1b1-/-* cells exhibited no detectable transcript for *Cyp1b1*. Although the level of VEGF mRNA was lower in *Cyp1b1-/-* LSEC it was not significantly different from *Cyp1b1*+/+ LSEC (Fig 4D). Thus, the decrease in VEGF level detected in Fig 4B is likely due to posttranslational mechanisms.

LSEC produce BMP6 that acts in a paracrine manner on liver hepatocytes to drive hepcidin expression in response to changes in systemic iron levels. BMP6 acting coordinately with hemojuvelin, a BMP6 co-receptor, to induce hepcidin production. Hepcidin interacts with the iron exporter protein ferroportin enhancing its turnover and decreasing iron release into plasma [15]. Fig 4E shows a significant decrease in the level of BMP6 produced by *Cyp1b1-/-* LSEC. These results are consistent with decreased expression of hepcidin reported in the livers from Cyp1b1-/- mice [37]. Thus, lack of *Cyp1b1* may induce a systemic iron overload resulting in increased oxidative stress and tissue damage, as we previously demonstrated [33, 35, 51].

Caveolin-1 is a structural protein in LSEC and closely related to autophagy. VEGF mediated PI3K-AKT-MTOR pathway and enhanced NO dependent pathways prevents autophagy and degradation of caveolin-1, and maintains fenestrae and suppresses fibrosis phenotype of LSEC [52]. In addition, knockdown of caveolin-1 facilitated defenestration due to activation of the AMPK dependent autophagy. Furthermore, decreased eNOS and NO activity could promote autophagy and increase defenestration. We observed an increase in the levels of caveolin-1α isoform while the level of caveolin-1β isoform was decreased in *Cyp1b1-/-* LSEC (Fig 4F). The level of eNOS was also significantly decreased in *Cyp1b1-/-* LSEC (Fig 4F), as we had shown in retinal EC due to increased oxidative stress [34]. These results are consistent with lack and/or decreased fenestration observed in *Cy1b1*-/- LSEC.

### Expression and organization of junctional proteins in LSEC

**Fig 5:**
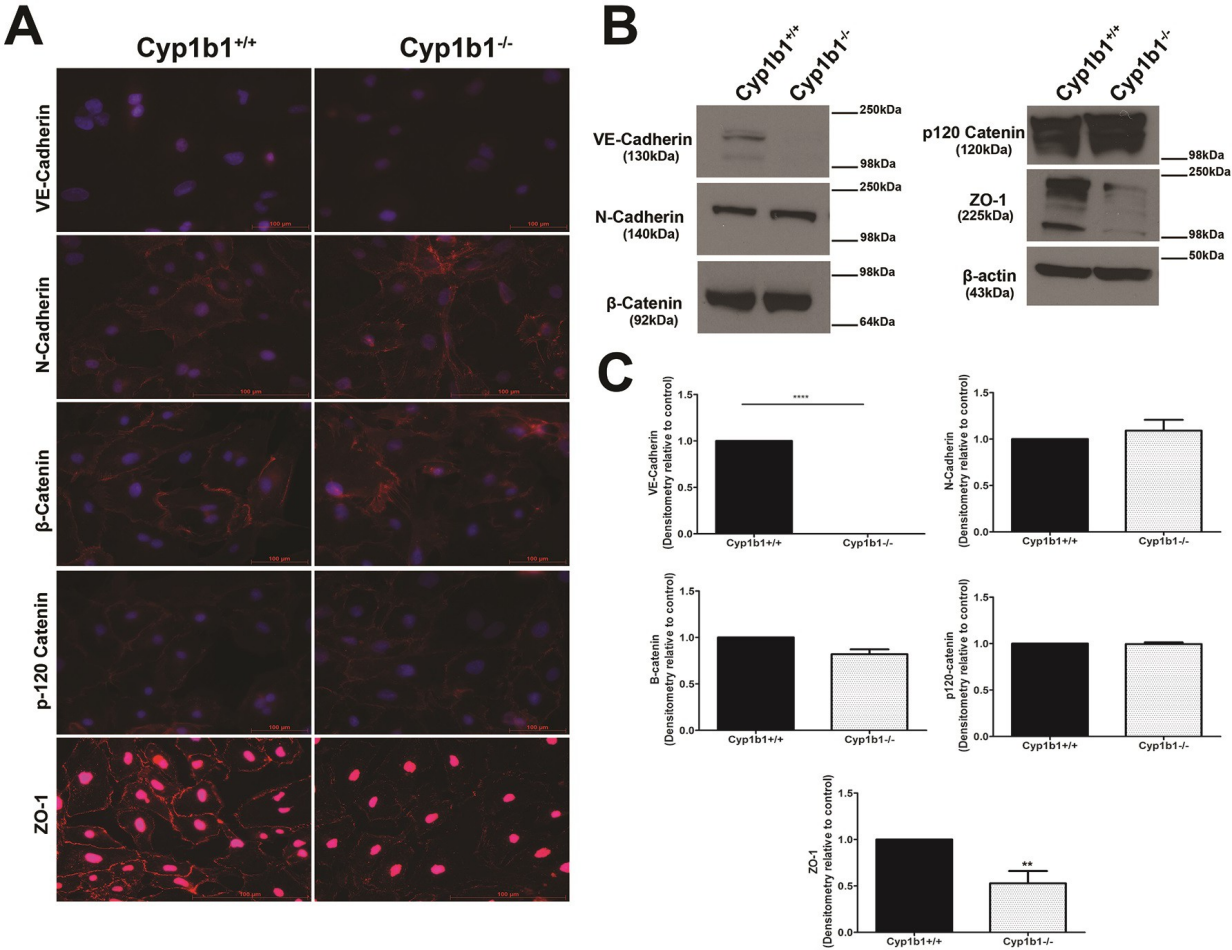
Expression and localization of junctional proteins. The junctional localization of various proteins involved in formation of adherens junction and tight junctions were determined by immunofluorescence staining using specific antibodies to VE-cadherin, N-cadherin, β-catenin, p120 catenin, and ZO-1 as detailed in Methods (A). The changes in the levels of these proteins were determined by Western blot analysis of cell lysates using specific antibodies (B). The quantitative assessment of the data is shown in (C). Please note a significant decrease in the levels of VE-cadherin and ZO-1 proteins in *Cyp1b1-/-* LSEC, while the levels of N-cadherin and p120 catenin were modestly increased (****P< 0.0001 and **P< 0.01; n = 3). These results are consistent with the intensity of staining seen in (A). These experiments were repeated with two isolations of LSEC with similar results.

Previous studies have indicated lack of junctional proteins as a consequence of the absence of adherens junction between LSEC [6]. We next examine the localization and expression levels of junction proteins including VE-cadherin, N-cadherin, β-catenin, p120 catenin and ZO-1. With the exception of VE-cadherin, the staining of all the proteins showed some junctional localization, specially N-cadherin, β-catenin, p120 catenin, and ZO-1 (Fig 5A). The staining levels were proportional to the protein levels examined by western blot analysis. A dramatic decrease in the levels of VE-cadherin and ZO-1 proteins was observed in *Cyp1b1-/-* LSEC, while the levels of P120 catenin was increased (Fig 5B). The decreased level of VE-cadherin is consistent with our inability to detect its junctional localization, especially in *Cyp1b1-/-* LSEC. This could be also partially attributed to the lack/reduced adherens junction formation in LSEC, and to the different sensitivity of the antibodies used in FACS and Western blot analysis. The FACS and Western blot analysis showed detectable levels of VE-cadherin in these cells, although at significantly lower levels in Cyp1b1-/- LSEC (Figs 1B and 5).

### Enhanced proliferation of *Cyp1b1-/-* LSEC

**Fig 6:**
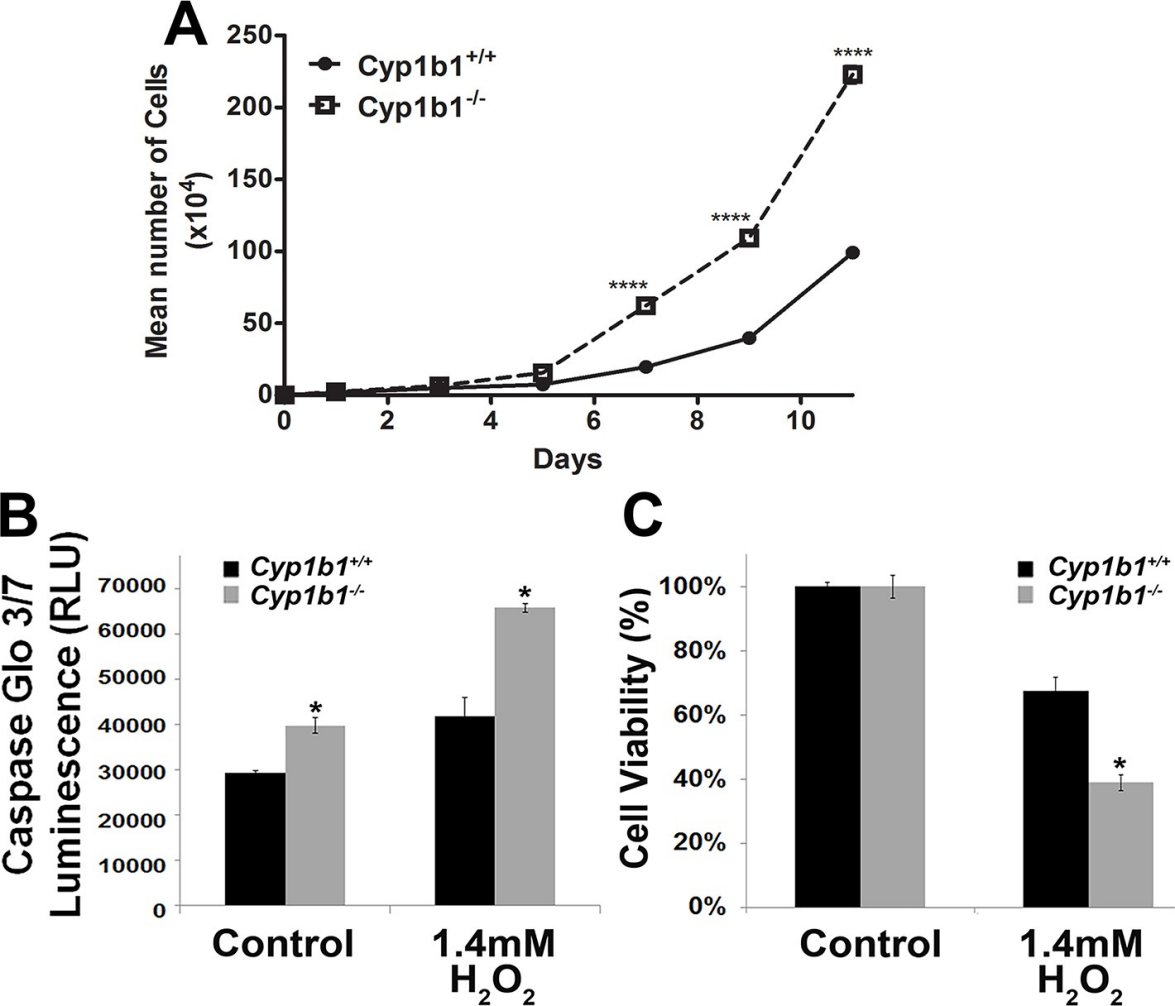
Increased rate of proliferation and apoptosis in *Cyp1b1-/-* LSEC. (A) The rate of proliferation was determined by counting the number of cells for 12 days. Please note a significant increase in the rate of *Cyp1b1-/-* LSEC proliferation. Enhanced apoptosis (B) and decreased viability (C) of *Cyp1b1-/-* LSEC in response to stress. Rate of apoptosis was determined by measuring caspase activity with luminescent signal from caspase-3/7 DEVD-aminoluciferin substrate. As an apoptosis stimulus, H_2_O_2_ in EC growth media was added for 8 h. Please note the significant increase in the rate of apoptosis in *Cyp1b1-/-* LSEC. Hydrogen peroxide (H_2_O_2_) toxicity of LSEC was determined with the 3-(4,5-dimethylthiazol-2-yl)-5-(3-carboxy-methoxyphenyl)2-(4-sulfophenyl)-2*H*-tetrazolium (MTS) assay method. LSEC were incubated with 1.4 Mm H_2_O_2_ in EC growth media for 24 h, and the level of toxicity was determined by measuring colorimetric change of MTS in cells on 96-well plates. *Cyp1b1-/-* liver EC were significantly less viable under the stress of H_2_O_2_ (*P<0.05; n = 3). These experiments were repeated with two isolations of LSEC with similar results.

We next determined the rate of proliferation in *Cyp1b1*+/+ and *Cyp1b1-/-* LSEC by counting the number of cells for two weeks. Fig 6A shows a significant enhancement in the rate of *Cyp1b1-/-* LSEC proliferation. We next asked whether the rate of apoptosis is different in these cells. The rates of apoptosis in *Cyp1b1+/+* and *Cyp1b1-/-* LSEC, both at steady-state and under exogenous stress, were determined by evaluation of the activation status of caspase 3/7. *Cyp1b1-/-* LSEC were found to be 25% more apoptotic than *Cyp1b1+/+* cells at the basal level. When incubated with 1.4 mM hydrogen peroxide (H_2_O_2_) for 8 h, *Cyp1b1-/-* LSEC were 40% more apoptotic than *Cyp1b1+/+* cells (Fig 6B).

The cell viability was evaluated with the MTS cytotoxicity assay. *Cyp1b1+/+* and *Cyp1b1-/-* LSEC were plated on a gelatin-coated 96-well plate and incubated with different concentration of H_2_O_2_ (0–2 mM) for 24 h. Cell viability was decreased in a concentration-dependent manner in both *Cyp1b1+/+* and *Cyp1b1-/-* LSEC (not shown). Specifically, incubation with 1.4 mM H_2_O_2_ decreased the viability of *Cyp1b1+/+* LSEC by 30%, while the viability of *Cyp1b1-/-* cells was decreased by 63% (Fig 6C). Thus, *Cyp1b1-/-* LSEC were more sensitive to H_2_O_2_-mediated cytotoxicity than *Cyp1b1+/+* cells. This is also consistent with the increased rate of apoptosis observed in *Cyp1b1-/-* LSEC under stress (Fig 6B).

### Cyp1b1-/- LSEC exhibit altered migratory and adhesive properties

**Fig 7:**
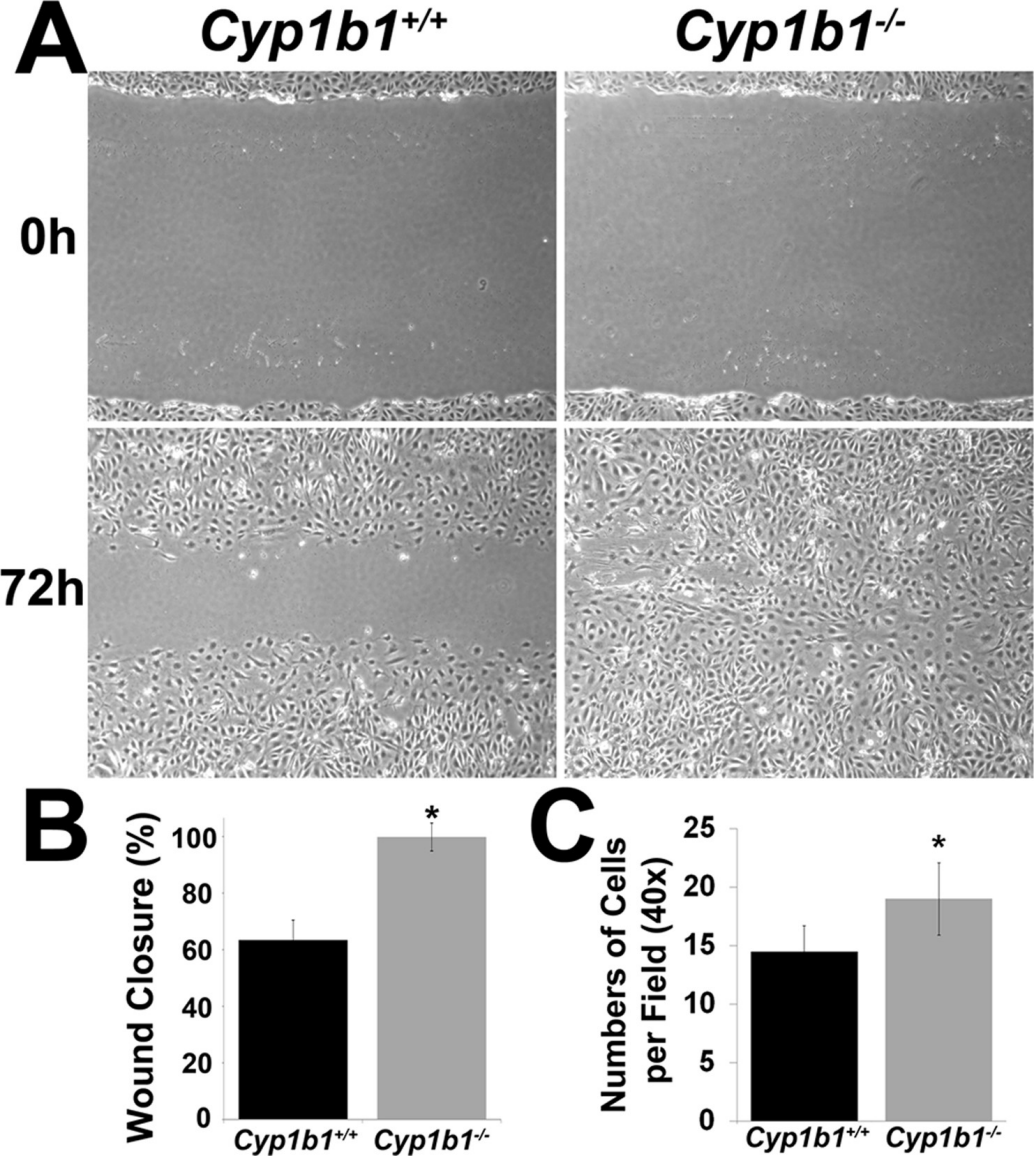
Cyp1b1 -/- LSEC are more migratory. (A) Confluent monolayers of *Cyp1b1+/+* and *Cyp1b1-/-* LSEC were wounded and wound closure was monitored and photographed for 72 h. Please note that a significant area of wound remained uncovered in *Cyp1b1*+/+ LSEC, while *Cyp1b1-/-* cells migrated and covered the wound. (B) Quantification assessment of the scratch wound assay. (C) *Cyp1b1-/-* LSEC were also more migratory than *Cyp1b1*+/+ cells in transwell migration assays (*P< 0.05; n = 3). These experiments were repeated with two isolations of LSEC with similar results.

Endothelial cell migration is fundamental to angiogenesis. We next examined the migratory properties of *Cyp1b1+/+* and *Cyp1b1-/-* LSEC by scratch wound assay. Confluent monolayers of *Cyp1b1+/+* and *Cyp1b1-/-* cells were wounded, and the wound closure by cell migration was monitored with still photography. As a result, a significant area of wound was still open in *Cyp1b1+/+* LSEC at 72 h. However, the wound was completely closed in the *Cyp1b1-/-* LSEC monolayer (Fig 7A). The quantitative assessment of the data is shown in Fig 7B. The enhanced migration of *Cyp1b1-/-* LSEC was also confirmed by transwell migration assays (Fig 7C).

**Fig 8:**
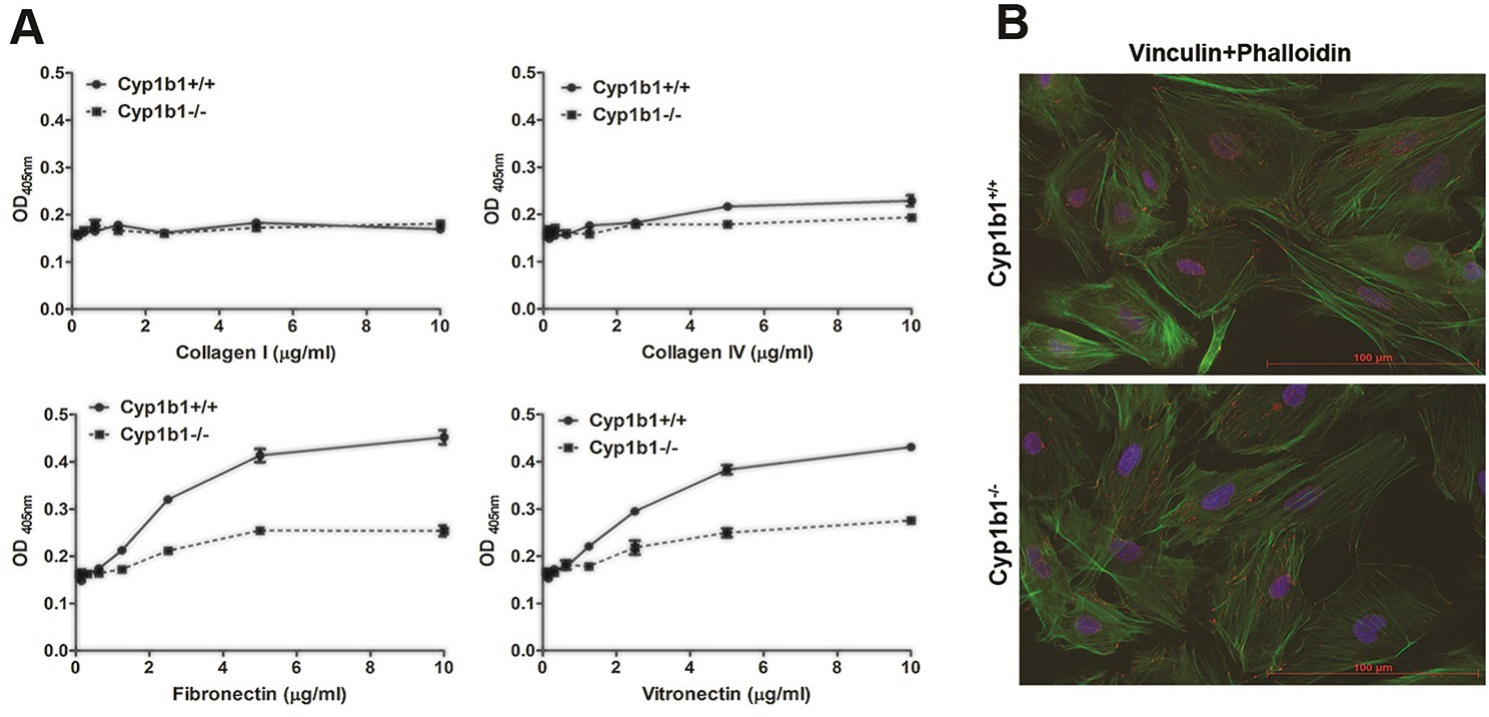
*Cyp1b1* -/- LSEC are less adherent to ECM proteins. (A) The adhesion of *Cyp1b1*+/+ and *Cyp1b1* -/- cells to various ECM proteins including fibronectin, vitronectin, collagen I and Collagen IV was determined as detailed in Methods. Please note that *Cyp1b1-/-* LSEC were less adhesive on fibronectin and vitronectin compared with *Cyp1b1*+/+ cells. (B) Immunofluorescence staining of *Cyp1b1*+/+ and *Cyp1b1-/-* LSEC with phalloidin (actin) and vinculin (focal adhesions). Please note increased actin stress fibers and peripheral organization of focal adhesions in *Cyp1b1-/-* LSEC, consistent with their enhanced migratory activity, compared with wild type cells. These experiments were repeated with two isolations of LSEC with similar results.

The alteration in migration of *Cyp1b1-/-* LSEC suggested alterations in cell adhesive mechanisms may exist. We next investigated the adhesion of *Cyp1b1+/+* and *Cyp1b1-/-* LSEC to various ECM proteins. Fig 8A shows the adhesion of *Cyp1b1-/-* LSEC to fibronectin, vitronectin, collagen I, and collagen IV. *Cyp1b1-/-* LSEC were significantly less adherent on fibronectin and vitronectin compared with *Cyp1b1*+/+ cells. These cells exhibited similar adhesion on collagen I and collagen IV. Fig 8B shows phalloidin and vinculin staining of these cells. *Cyp1b1-/-* LSEC showed more prominent actin stress fibers and peripheral focal adhesions consistent with their enhanced migratory characteristic. However, *Cyp1b1*+/+ cells were more spread with predominant peripheral actin staining and basally distributed focal adhesions consistent with their more adherent characteristic.

### Alterations in ECM proteins and their receptors in *Cyp1b1-/-* LSEC

**Fig 9:**
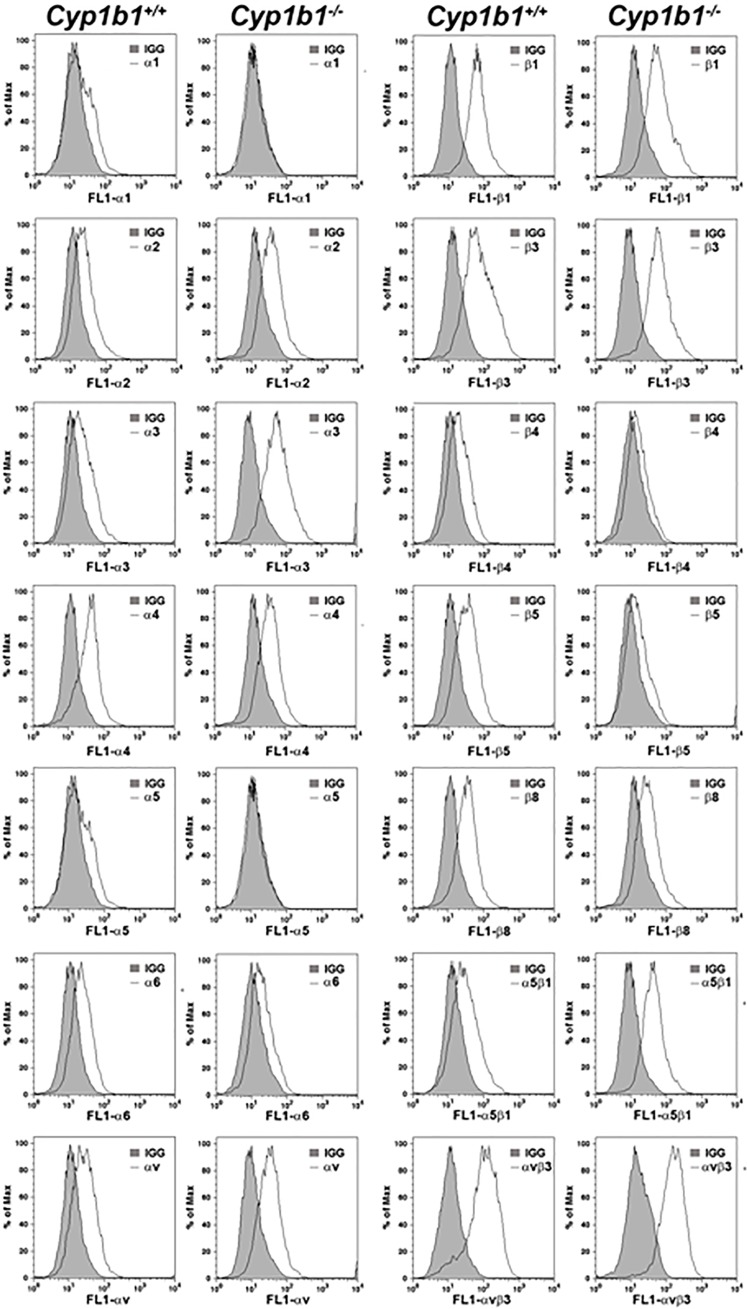
Altered expression of integrins in *Cyp1b1-/-* LSEC. The expression of various integrins in LSEC was determined by FACS analysis as detailed in Methods. *Cyp1b1-/-* LSEC expressed increased levels of α3, α5β1, and αvβ3. The levels of α1, α5, β4, β5, and β8 was decreased in *Cyp1b1-/-* cells compared with *Cyp1b1+/+* cells. These experiments were repeated with two isolations of LSEC with similar results.

The change in migratory and adhesive properties of LSEC suggested changes in expression and/or activity of integrins, the receptors for various ECM proteins. We next examined the expression of various integrins in *Cyp1b1*+/+ and *Cyp1b1-/-* LSEC by FACS analysis. These included α1, α2, α3, α4, α5, α6 and αv integrins. We also examined the expression of β1, β2, β4, β5, β8, α5β1, and αvβ3 integrins. These cells expressed similar levels of α2, α4, α6, αv, β1, β3 but *Cyp1b1-/-* cells expressed increased levels of α3, α5β1, and αvβ3. The levels of α1, α5, β4, β5, and β8 was decreased in *Cyp1b1-/-* cells compared to *Cyp1b1*+/+ cells (Fig 9). However, the changes in activity and avidity of these integrins cannot be ruled out.

**Fig 10:**
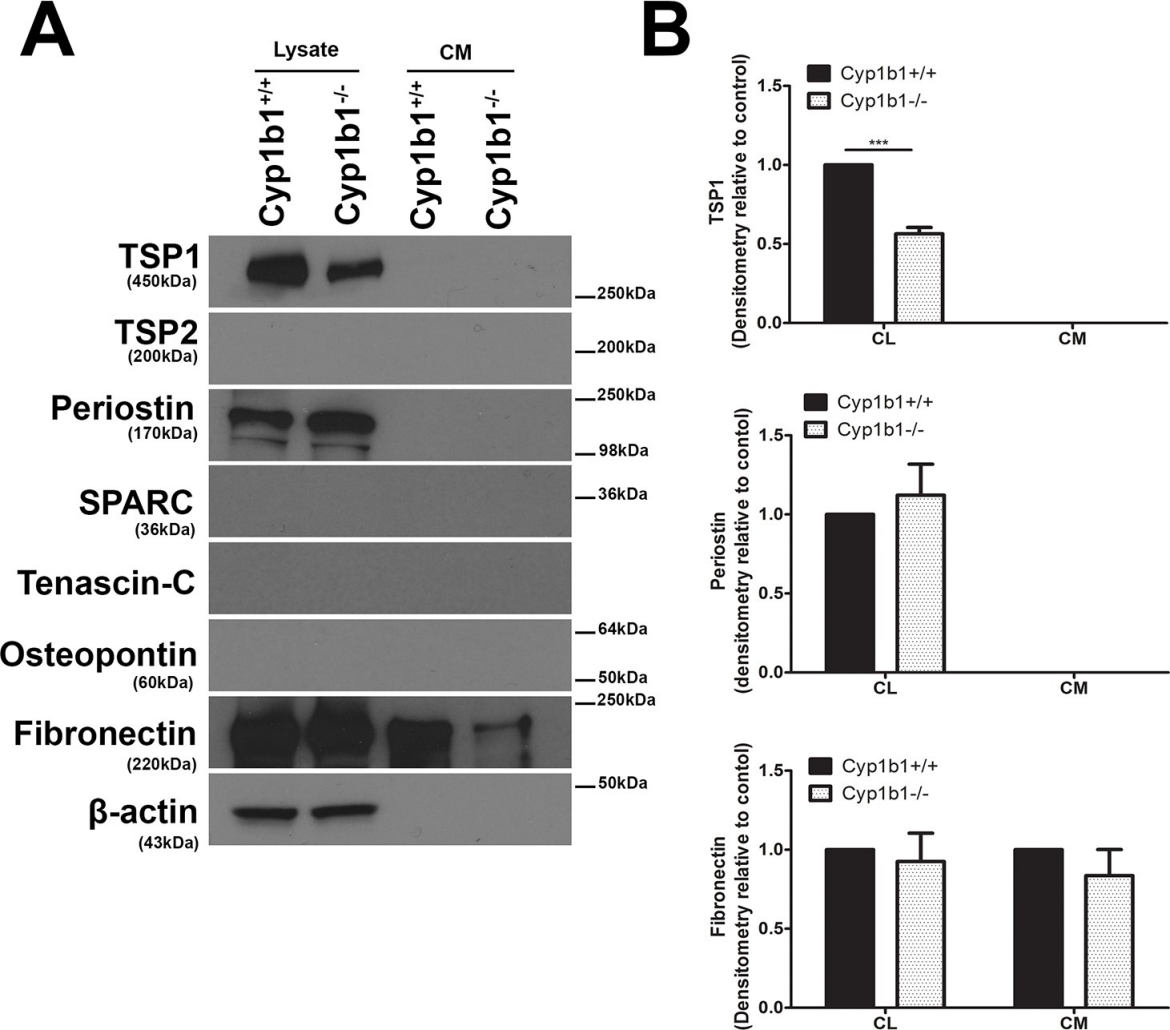
Altered expression of ECM proteins in *Cyp1b1-/-* LSEC. Western blot analysis of ECM proteins in the conditioned medium (CM) and cell lysates from LSEC was performed. (A) The levels of SPARC, Tenascin-C, TSP2, and Osteopontin were below the level of detection. *Cyp1b1* LSEC produced TSP1, periostin, and fibronectin. (B) The quantitative assessment of the data. TSP1 level was significantly decreased in lysates from *Cyp1b1-/-* LSEC (***P< 0.001; n = 3), while periostin level was increased in lysates from *Cyp1b1-/-* LSEC. The level of fibronectin secreted into the conditioned medium was decreased in *Cyp1b1-/-* LSEC. These experiments were repeated with two isolation of LSEC with similar results.

Changes in the proportions of ECM components of LSEC may also result in their altered adhesive and migratory properties, and impaired passage of lipoproteins across the sinusoids [53]. To further investigate this possibility, the ECM production profile of *Cyp1b1+/+* and *Cyp1b1-/-* LSEC, both in conditioned medium (CM) and cell lysates was determined. Fig 10 shows a significant decrease in the level of TSP1 in cell lysate prepared from *Cyp1b1-/-* LSEC compared with *Cyp1b1*+/+ cells. No TSP1 was detected in the conditioned medium collected from these cells. We also failed to detect TSP2, SPARC, tenascin C, and osteopontin in these cells. However, we observed an increase in the level of periostin produced by *Cyp1b1-/-* LSEC. These cells also expressed lower amount of fibronectin in their conditioned medium. The decreased levels of TSP1 in *Cyp1b1-/-* LSEC is consistent with increased migration and proliferation of these cells as we showed with TSP1-/- retinal EC [40, 54]

### Cyp1b1 expression minimally affects capillary morphogenesis of LSEC

**Fig 11:**
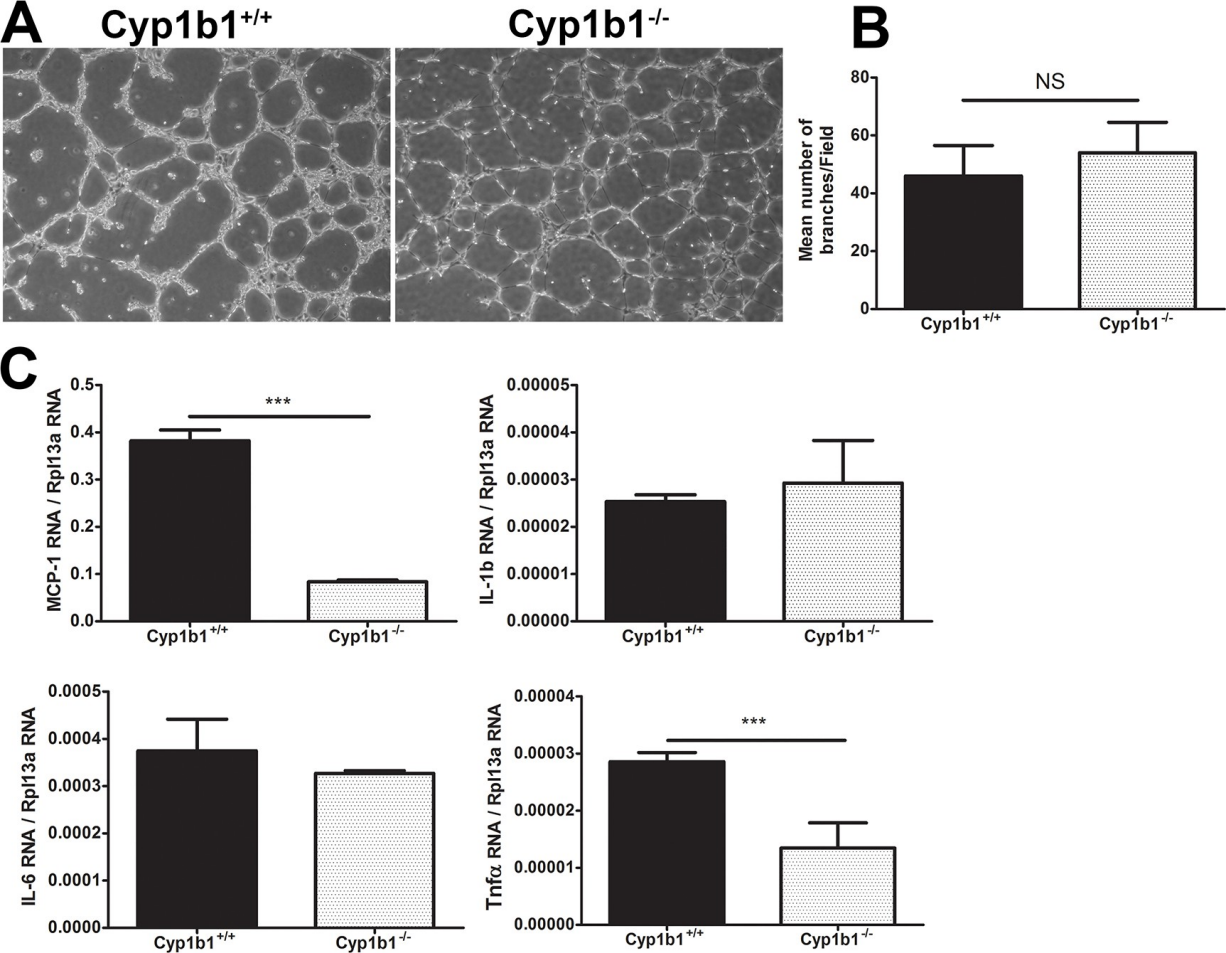
Capillary morphogenesis and expression of inflammatory mediators in LSEC. The capillary morphogenesis of *Cyp1b1*+/+ and *Cyp1b1-/-* LSEC were assessed by plating on Matrigel as detailed in methods (A). The quantitative assessment of the data is shown in (B) (P> 0.05; n = 3). Please note similar degree of branching but less cellular character of branched sprouts in *Cyp1b1-/-* LSEC. The expression of various inflammatory mediators including MCP-1, IL-1β, IL-6, and Tnf-α were determined by qPCR analysis. A significant decreases in the levels of MCP-1 and TNF-α was observed (C; ***P< 0.001; n = 3,). These experiments were repeated with two isolations of LSEC with similar results.

One of the characteristics of EC is their ability to undergo capillary morphogenesis when plated on Matrigel. This process is influenced by adhesive and migratory characteristics of EC. We next asked whether Cyp1b1 expression affects the ability of LSEC to undergo capillary morphogenesis in Matrigel. Fig 11A shows the ability of LSEC to undergo capillary morphogenesis is minimally affected by Cyp1b1 expression without a significate difference in the mean number of branches observed (Fig 11B). However, the networks formed by *Cyp1b1-/-* LSEC appeared to have fewer cells. This could be linked to the reduced levels of TSP1, an endogenous inhibitor of angiogenesis, and enhanced migration and reduced adhesion of *Cyp1b1-/-* LSEC.

We have shown that inflammatory mediators have adverse effects on EC [55]. We next examined the production of various inflammatory mediators including MCP-1, IL-1β, IL-6, and TNF-α. We observed a significant decrease in the amount of MCP-1 and TNF-α expressed in *Cyp1b1-/-* LSEC compared to *Cyp1b1*+/+ cells (Fig 11C). However, no significant differences was observed in levels of IL-1β and IL-6.

### Sustained activation of AKT and ERK signaling pathways in *Cyp1b1-/-* LSEC

**Fig 12:**
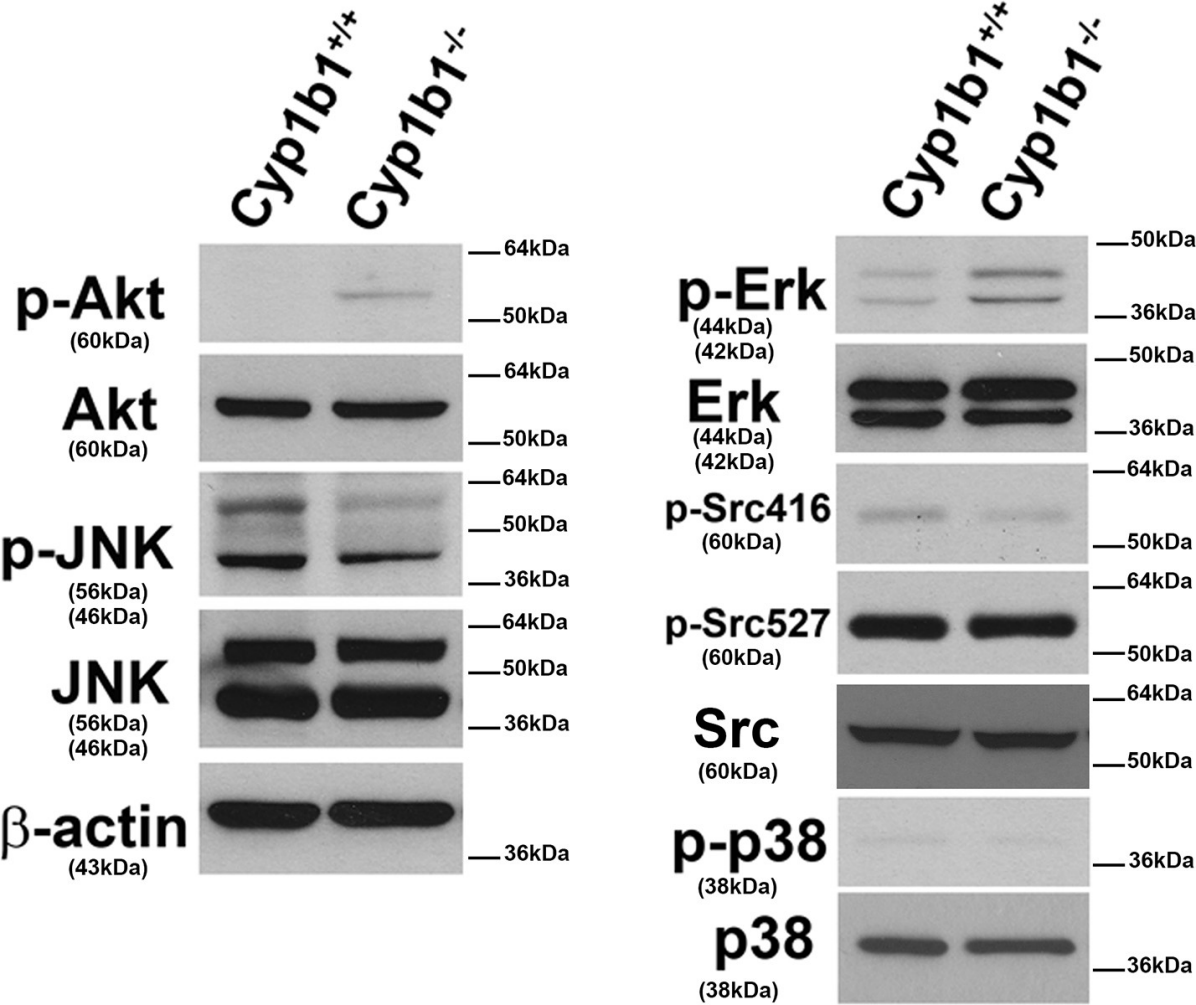
Sustained activation of AKT and ERKs signaling pathways in *Cyp1b1-/-* LSEC. The activation of various signaling pathways were examined by Western blot analysis of cell lysates prepared from *Cyp1b1*+/+ and *Cyp1b1-/-* LSEC as detailed in Methods. Please note an increase in the levels of phosphorylated AKT (p-AKT) and ERK (p-ERK), and a decrease in p-JNK in *Cyp1b1-/-* cells. The levels of phosphorylated Src (p-Src416 and p-Src527) and MAPK p38 (p-p38) were not affected. Similar levels of total proteins were observed in both cell types. These experiments were repeated with two isolations of LSEC with similar results.

The AKT/PKB and MAPK signaling pathways have significant impact on survival and proliferation of various cell types including EC. We next examined the activation status of AKT and MAPK pathways using specific antibodies for the active phosphorylated forms of these proteins as well as their total levels. Fig 12 shows enhanced levels of AKT and ERK phosphorylation, while levels of phosphorylated JNK was diminished in *Cyp1b1-/-* LSEC. The levels of phosphorylated Src and P38 were not affected. A similar levels of total proteins were detected for all the proteins examined. The sustained AKT and ERK activation in *Cyp1b1-/-* LSEC is consistent with their enhanced proliferation and migration.

## Discussion

We previously showed that *Cyp1b1* is constitutively expressed in vascular cells from various tissues, and that lack of *Cyp1b1* was associated with increased oxidative stress and attenuation of proangiogenic activity [33, 34, 36, 56]. Although constitutive expression of *Cyp1b1* is limited in hepatocytes [31, 32], we hypothesized that LSEC constitutively express *Cyp1b1* with significant impact on their inflammatory and angiogenic properties. Here we showed that LSEC constitutively expressed significant amount of Cyp1b1, which was absent in *Cyp1b1-/-* cells as expected. We showed LSEC express many of the markers expressed by EC from other vascular beds, as well as lymphatic markers VEGFR3, LYVE-1, and PROX-1 as previously reported [6]. Although we observed fenestrations in *Cyp1b1*+/+ LSEC, we were unable to detect such fenestration in *Cyp1b1-/-* cells perhaps as a result of reduced VEGF levels produced by these cells. *Cyp1b1-/-* cells showed minimal junctional localization of adherens and gap junction proteins, consistent with a dramatic decrease in the levels of VE-cadherin and ZO-1 in these cells. We also showed that *Cyp1b1-/-* LSEC proliferated at a faster rate and exhibited an enhanced rate of apoptosis, especially when challenged with H_2_O_2_. The *Cyp1b1-/-* LSEC were more migratory but less adherent compared with *Cyp1b1*+/+ cells. These changes were consistent with alterations observed in the expression of various integrins and ECM proteins in *Cyp1b1-/-* LSEC. The *Cyp1b1-/-* LSEC also showed decreased production of inflammatory mediators MCP-1 and TNF-α, and sustained activation of AKT and ERK signaling pathways. Thus, our data collectively suggest an important role for Cyp1b1 expression in modulation of angiogenic and inflammatory characteristic of LSEC, likely through modulation of cellular redox homeostasis [33, 35, 51].

A major characteristic of LSEC in vivo is their fenestration. This differentiated LSEC phenotype is maintained by autocrine and paracrine regulation [57]. The VEGF-mediated paracrine effect of hepatocytes or stellate cells is essential for mainiaing the differentiated phenotype LSEC. Capillarization is a phenotype of LSEC preceding fibrosis, which is associated with increased expression of PECAM-1/CD31, VCAM-1, and loss of fenestration [58]. Here we observed that the LSEC express significant amount of these proteins and also appear to have less fenestration, especially in *Cyp1b1-/-* cells. In addition, LSEC normally express low levels of VE-cadherin, due to lack of adherens and other types of junctions in these cells [6]. Although our FACS data showed significant expression of VE-cadherin on the surface of LSEC, our Western blot analysis showed a dramatic decrease in the levels of VE-cadherin and ZO-1 in *Cyp1b1* -/- LSEC. An increase in VE-cadherin and ICAM-1 level was reported in LSEC under chronic inflammation [6, 59]. The decreased levels of VE-cadherin, ZO-1, and ICAM-1 in *Cyp1b1-/-* LSEC observed here is consistent with reduced expression of inflammatory mediators MCP-1 and TNF-α in these cells. In addition, we previously showed sustained activation of MAPK/ERKs results in decreased expression of EC adhesion molecules including PECAM-1 and VE-cadherin [60]. Furthermore, decreased levels of VEGF by these cells may contribute to their increased loss of fenestration. These results are also consistent with increased oxidative stress and sensitivity of *Cyp1b1-/-* LSEC to oxidative challenge. However, the identity of exact mechanisms involved remain elusive and are subject of future investigation.

A major characteristic of EC is their ability to respond to proangiogenic signals by enhanced migration, proliferation, and differentiate into capillary networks. We found that *Cyp1b1-/-* LSEC proliferated and migrated at a faster rate and were less adherent when compared to *Cyp1b1*+/+ cells. We did not note significant differences in the ability of these cells to undergo capillary morphogenesis. However, the networks formed by *Cyp1b1-/-* LSEC had significantly smaller islands of cells and number of cells aligned with the cord networks. This could be attributed to the enhanced migratory and reduced adhesive properties of these cells. These observations are consistent with our previous report of the ability of TSP1-/- retinal EC, which are more proliferative and migratory, and similarly form capillary networks that are less cellular [40]. Here we showed that *Cyp1b1-/-* LSEC do produce less TSP1 compared with *Cyp1b1*+/+ cells. In addition, TSP1-deficient EC also exhibit enhanced proliferation and sustained activation of MAPK/ERKs [54], as was also demonstrated here for *Cyp1b1-/-* LSEC. However, a peptide from C-terminal domain of TSP1 that binds CD47 was shown to induce defenestration of LSEC [47]. Thus, the role of TSP1 and its peptides in modulation of LSEC function and fenestration needs further investigation.

The production of BMP6 by LSEC plays a key role in systemic iron hemostasis through its paracrine action on liver hepatocytes and modulation of hepcidin production [15, 61]. Hepcidin is a key regulator of iron absorption and binds the cellular iron exporter ferroportin inducing its endocytosis and proteolysis. In this way, it prevents iron release from macrophages or intestinal cells into the plasma. Hepcidin deficiency results in excessive iron absorption from diet and deposition of iron in liver and other tissues [61]. The increased iron levels result in enhanced expression of BMP6 in LSEC, which enhances hepcidin expression in hepatocytes to attenuate increased systemic iron levels [62]. The absence of BMP6 in the mice results in a rapid and massive accumulation of iron in the liver and other tissues due to diminished levels of hepcidin [15]. We observed decreased levels of BMP6 produced by *Cyp1b1-/-* LSEC compared with *Cyp1b1*+/+ cells. This is consistent with reported decreased hepcidin expression in the livers of *Cyp1b1-/-* mice [37]. Thus, *Cyp1b1* deficiency may lead to enhanced systemic iron levels and increased iron accumulation in various tissues. The increase iron levels results in increased oxidative stress due to iron mediated Fenton reaction and production of hydroxy radicals, which are very reactive, and enhance lipid peroxidation and oxidative stress [63]. We previously reported enhanced levels of lipid peroxidation and formation of 4-HNE adducts in eyes of *Cyp1b1-/-* mice subjected to OIR compared to wild type mice, which was elevated by treatment of animals with the anti-oxidant N-acetylcysteine, a major ROS scavenger [33]. Thus, altered iron homeostasis in response to Cyp1b1-deficiency may significantly impact tissue redox homeostasis and developmental processes linked to Cyp1b1 expression including the trabecular meshwork and pathogenesis of primary congenital glaucoma [64].

In summary, the expression of Cyp1b1 is essential for modulation of the angiogenic and inflammatory properties of LSEC. *Cyp1b1-/-* LSEC proliferated and migrated at a faster rate and were less adherent. In addition, the expression of Cyp1b1 in LSEC maintains the basal inflammatory characteristics of these cells and its absence resulted in decreased production of inflammatory mediators. Furthermore, decreased production VEGF by *Cyp1b1-/-* LSEC may promote their defenestration. However, decreased levels of VE-cadherin and ZO-1, and reduced formation of cell-cell junctions is consistent with the differentiated phenotype of *Cyp1b1-/-* LSEC and deserve further investigation.
